# Pilot Mixed-Methods Evaluation of Simulation-Based Focused Cardiac Ultrasound Teaching for Final-Year Medical Students

**DOI:** 10.7759/cureus.117192

**Published:** 2026-09-30

**Authors:** Vishnuga Uthayamohan, Katie Allen, Olalekan Ogundana, Saad Abdulla

**Affiliations:** 1 Emergency Medicine, Basildon and Thurrock University Hospitals NHS Foundation Trust, London, GBR; 2 Emergency Medicine, Basildon and Thurrock University Hospitals NHS Foundation Trust, Basildon, GBR

**Keywords:** bedside echocardiography, clinical education, emergency medicine, medical students, point-of-care ultrasound (pocus), simulation-based education, ultrasound education, undergraduate medical education

## Abstract

Background

Point-of-care ultrasound (POCUS) is increasingly used within emergency and acute care, yet undergraduate exposure to focused bedside echocardiography remains variable. Simulation may provide an opportunity for students to develop practical ultrasound skills while integrating findings into clinical assessment and decision-making. This study evaluated the feasibility and acceptability of a simulation-based bedside echocardiography teaching programme for final-year medical students and explored differences in confidence and knowledge between pre- and post-session respondents.

Methodology

A mixed-methods educational evaluation was undertaken with final-year medical students during their Preparation for Practice placement at a UK teaching hospital. The two-hour programme combined teaching on ultrasound principles and standard echocardiographic views with supervised image acquisition and simulation-based peri-arrest scenarios involving cardiac arrest, massive pulmonary embolism and pericardial tamponade. Thirty students were invited and 10 attended. Anonymous questionnaires assessed self-reported confidence and knowledge immediately before and after the session, and a five-question multiple-choice quiz objectively assessed knowledge. We also collected qualitative free-text feedback post-session. Confidence reflected participants’ own perceived confidence across three domains of bedside echocardiography and was not assessed externally or used as a measure of objective competence. As questionnaires were anonymous, pre- and post-session responses were unpaired. Self-reported confidence scores were compared using the Mann-Whitney U test.

Results

Nine students completed the pre-session questionnaire, and seven completed the post-session questionnaire. Mean confidence scores were higher among post-session respondents (9.86/15; median, 10) than among pre-session respondents (3.67/15; median, 4), with a statistically significant difference between groups (*U* = 0.0, *P* < 0.001). Mean knowledge scores were also higher among post-session respondents (4.71/5 vs. 4.22/5), although baseline scores were already high. Qualitative feedback was positive, with students particularly valuing the clinical relevance of the teaching and the integration of echocardiography into realistic emergency scenarios. Participants identified additional supervised scanning opportunities as an area for future development.

Conclusions

This preliminary evaluation suggests that a short, simulation-based bedside echocardiography teaching programme is feasible to deliver within an undergraduate prep-for-practice placement and acceptable to participating final-year medical students. Higher confidence and knowledge scores were observed among post-session respondents; however, the small sample size and unpaired questionnaire design prevent conclusions regarding individual learner improvement or intervention efficacy. Larger studies incorporating paired responses, objective assessment of ultrasound competence and longitudinal follow-up are required to evaluate the effectiveness and retention of this educational approach.

## Introduction

Point-of-care ultrasound (POCUS) has become an increasingly important diagnostic and procedural tool across a wide range of acute medical specialities, including emergency medicine, intensive care and acute internal medicine. Focused bedside echocardiography (Echo), as part of POCUS, enables clinicians to rapidly assess life-threatening cardiac pathology such as pericardial effusion, severe left ventricular dysfunction, right ventricular strain secondary to pulmonary embolism and profound hypovolaemia. In emergency settings, these findings can influence immediate clinical decision-making and guide resuscitation alongside conventional clinical assessment [1]. With ultrasound technology becoming increasingly portable and accessible, POCUS, including focused echocardiography, is increasingly embedded within the assessment of critically unwell patients. A key advantage of POCUS is that imaging is performed and interpreted at the bedside by the treating clinician, allowing findings to be interpreted in conjunction with the patient’s clinical presentation and incorporated immediately into ongoing assessment and management. This is particularly valuable at initial presentation, following clinical deterioration, or when patients are too unstable for formal departmental imaging.

Recognising this shift in clinical practice, professional organisations have increasingly advocated the integration of ultrasound education within undergraduate medical curricula. The General Medical Council (GMC) emphasises that newly qualified doctors should be able to assess acutely unwell patients, make appropriate clinical decisions and utilise diagnostic investigations safely and effectively [2]. Although the GMC does not mandate competency in bedside echocardiography specifically, the growing use of POCUS across emergency and acute care has prompted many medical schools to introduce ultrasound teaching in various forms. However, there remains considerable variation in both the quantity and quality of undergraduate ultrasound education. Some institutions provide longitudinal ultrasound programmes throughout medical school, whereas others offer only isolated teaching sessions or no formal exposure at all [3]. Consequently, many graduates report limited confidence in performing or interpreting focused bedside ultrasound before commencing Foundation training.

Previous studies have demonstrated that undergraduate ultrasound teaching can improve anatomical understanding, procedural confidence and image interpretation skills [3,4]. However, much of the published literature has focused on ultrasound as a tool for teaching anatomy or isolated technical image acquisition rather than its application within authentic clinical decision-making. Final-year medical students approaching graduation require opportunities to integrate technical skills with clinical reasoning, communication and patient assessment to support their transition into clinical practice. Teaching focused bedside echocardiography within realistic emergency scenarios may therefore better reflect the context in which these skills are ultimately used.

Simulation-based education offers an established approach for developing clinical competence in psychologically safe learning environments. By recreating realistic clinical situations without risk to patients, simulation allows learners to practise technical procedures alongside non-technical skills including clinical reasoning, teamwork and decision-making. Kolb's experiential learning theory proposes that knowledge develops through experience, reflection and application [5]. Simulation supports this cycle by allowing learners to apply new knowledge in realistic scenarios and subsequently in patient-facing settings. Situated learning similarly emphasises learning within authentic clinical contexts [6]. Delivering POCUS Echo teaching through emergency or intensive care clinicians may strengthen this relevance, as these professionals will support students as they transition into Foundation training and can integrate technical skills with clinically relevant debriefing and decision-making.

Although POCUS is increasingly incorporated into undergraduate medical education, evidence evaluating bedside echocardiography teaching during the transition to Foundation training remains limited. At Basildon Hospital, final-year students undertake a one-month Preparation for Practice placement designed to prepare them for the practical realities of working as an FY1, including assessment of acutely unwell patients and clinical decision-making. Following a pilot needs assessment, a simulation-based bedside echocardiography programme was developed to allow students to apply focused cardiac ultrasound within clinically relevant peri-arrest scenarios, supporting the transition from medical student to Foundation doctor.

This study aimed primarily to evaluate the feasibility and acceptability of a simulation-based bedside echocardiography teaching programme for final-year medical students, while exploring differences in self-reported confidence and objectively assessed knowledge between pre- and post-session respondents.

## Materials and methods

Study design

This study evaluated a simulation-based bedside echocardiography teaching programme delivered to final-year medical students undertaking their Preparation for Practice placement at Basildon Hospital, United Kingdom. A mixed-methods educational evaluation was undertaken using pre- and post-session questionnaires to assess changes in students' knowledge and confidence, alongside qualitative feedback exploring learners' perceptions of the teaching programme.

Setting and participants

The teaching programme was offered to all final-year medical students allocated to the Preparation for Practice placement, a post-finals clinical module designed to support the transition from medical student to Foundation Year 1 (FY1) doctor by preparing students for the practical responsibilities of clinical practice. A total of 30 students were invited to participate in the voluntary two-hour teaching session, with 10 students attending. Attendance was entirely optional and did not contribute towards formal undergraduate assessment.

Prior to programme development, a pilot teaching session involving five final-year medical students was undertaken as a needs assessment. Feedback from this pilot demonstrated that students had very limited previous experience of bedside echocardiography and highlighted a preference for clinically relevant, simulation-based learning rather than traditional didactic teaching alone. The pilot informed refinement of the final teaching programme, including the incorporation of high-fidelity emergency scenarios and increased opportunities for hands-on practice.

Educational intervention

The teaching programme was designed and delivered by an experienced Emergency Medicine faculty comprising an Emergency Medicine consultant, registrars and Foundation Year 2 (FY2) doctor, with collective experience in point-of-care ultrasound, resuscitation and medical education.

The overall aim was to introduce the principles of POCUS Echo and enable students to apply these skills within common emergency presentations encountered by Foundation Year doctors. Learning objectives included understanding the basic principles of ultrasound, acquiring standard echocardiographic views, recognising clinically significant findings and integrating bedside echocardiography into the assessment of an acutely unwell patient. The programme consisted of 2 one-hour sessions delivered in small groups.

The first hour introduced the fundamental principles of ultrasound, including image generation, probe orientation, machine optimisation, and indications for focused bedside echocardiography in emergency medicine. Teaching was undertaken using a bedside ultrasound machine with a cardiac probe, allowing students to practise probe handling and image acquisition under supervision. Students were introduced to the focused cardiac examination and taught four standard echocardiographic views: parasternal long-axis, parasternal short-axis, apical four-chamber, and subcostal views. Teaching focused on recognising clinically significant pathology relevant to emergency practice, including pericardial effusion, right ventricular dilatation suggestive of massive pulmonary embolism, and severe left ventricular impairment.

The second hour comprised simulation-based teaching using realistic emergency scenarios involving cardiac arrest, massive pulmonary embolism, and pericardial tamponade. Students managed simulated peri-arrest patients using a structured A-E assessment while integrating bedside echocardiography into their clinical assessment, interpretation, and subsequent decision-making.

Following each scenario, learners participated in a structured, faculty-led debrief. Students were encouraged to reflect on their assessment and clinical decision-making before faculty facilitated discussion of the ultrasound findings, clinical management, and technical and non-technical aspects of performance. This included communication, clinical reasoning, and appropriate integration of POCUS Echo into the assessment of the acutely unwell patient. The structure of the educational intervention and evaluation is summarised in Figure 1.

**Figure 1:**
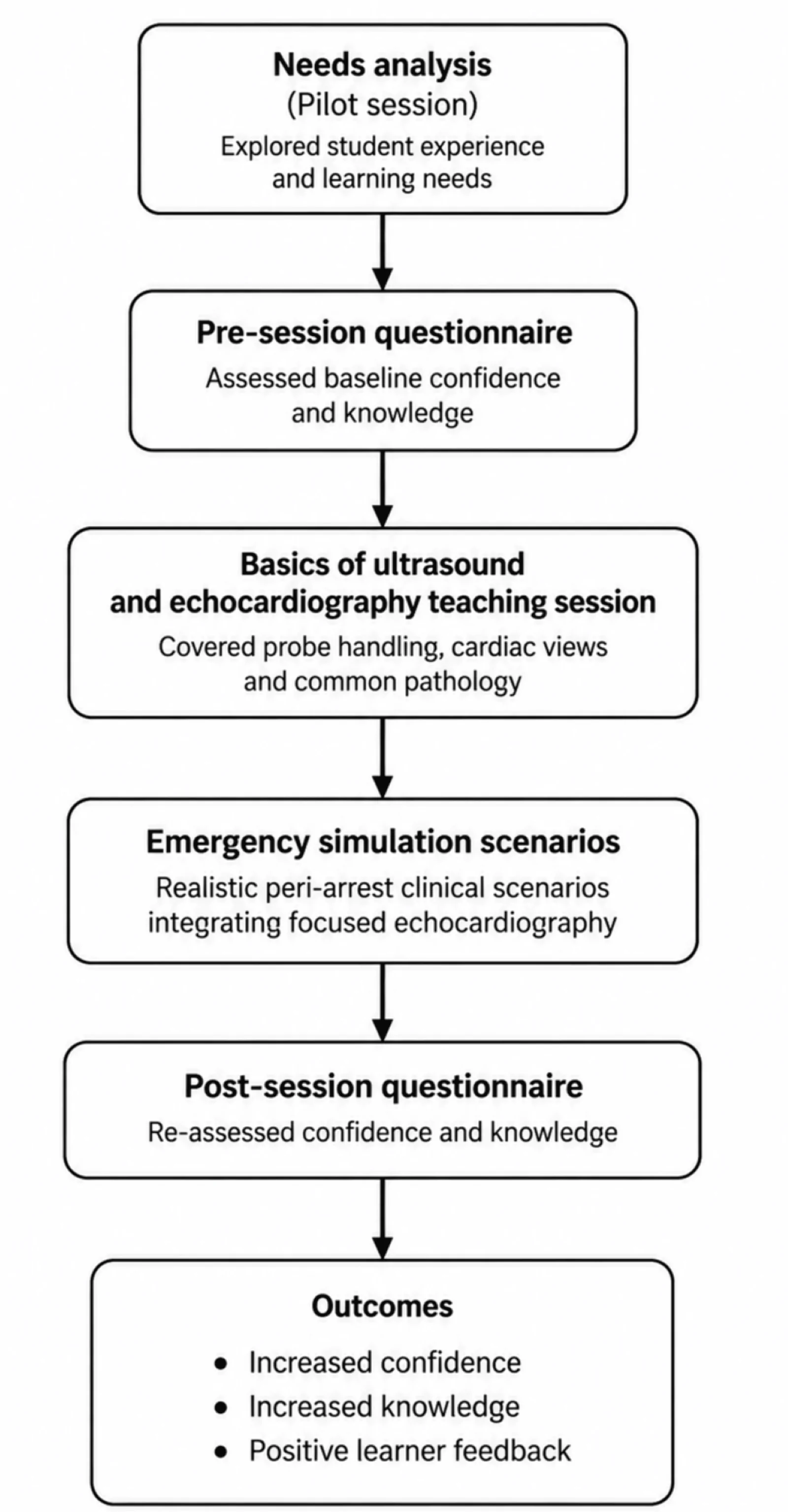
Overview of the simulation-based bedside echocardiography teaching programme and evaluation process. This image was created by the authors using Microsoft Word (Microsoft Corporation, Redmond, WA), using the built-in Shapes and Text tools.

Outcome measures

Participants completed anonymous questionnaires immediately before and after the teaching session.

The pre-session questionnaire assessed participants’ previous exposure to bedside echocardiography and their self-reported confidence across three domains: recognising life-threatening echocardiographic findings, performing probe positioning for standard cardiac views, and understanding basic cardiac ultrasound views. Each domain was rated on a five-point scale from 1 (not confident) to 5 (very confident), giving a total confidence score ranging from 3 to 15. Participants were also asked about the clinical situations in which they considered bedside echocardiography to be indicated and what they hoped to gain from the session.

Knowledge was objectively assessed using a five-question multiple-choice quiz covering the clinical applications and limitations of bedside echocardiography, recognition of pericardial effusion using the subcostal view, and the role of bedside echocardiography during cardiac arrest. Each correct answer was awarded one point, producing a total knowledge score ranging from 0 to 5.

The post-session questionnaire repeated the three confidence questions and the five-question knowledge assessment. Participants were additionally asked to identify three things they had learnt during the session and to evaluate the delivery of the programme across three domains: clarity of learning objectives, engagement with the teaching methods, and appropriateness of session pacing. Each aspect of session delivery was rated on a five-point scale from 1 (strongly disagree) to 5 (strongly agree). An open-ended question invited participants to provide additional feedback and suggestions for improvement.

The complete pre- and post-session questionnaires are provided in Appendices A-B.

Data analysis

Questionnaire data were exported into Microsoft Excel for analysis. As pre- and post-session questionnaires were completed anonymously, responses could not be paired. Descriptive statistics were calculated for participant characteristics, confidence scores and knowledge assessment results. Continuous variables are presented as means, medians and ranges, where appropriate.

Differences in confidence scores between pre- and post-session questionnaires were analysed using the Mann-Whitney U test owing to the small sample size and non-parametric distribution of unpaired data. Statistical significance was defined as a *P*-value of <0.05.

Free-text responses were analysed using an inductive thematic approach. Responses were independently reviewed, coded according to recurring concepts and grouped into overarching themes describing learners’ experiences of the programme and recommendations for future implementation.

Ethical considerations and governance

This project was undertaken as an evaluation of an educational intervention involving final-year medical students. Participation in both the teaching session and questionnaire evaluation was voluntary, and questionnaire responses were collected anonymously. No identifiable participant or patient data were collected. As the project constituted an evaluation of an existing educational activity, formal research ethics committee approval was not required. Completion of the questionnaires was taken to indicate consent to participate in the evaluation.

## Results

Participant characteristics

Thirty final-year medical students undertaking their Preparation for Practice placement were invited to participate in the teaching programme, of whom ten attended the session. Nine students completed the pre-session questionnaire, and seven completed the post-session questionnaire. As the questionnaires were completed anonymously, responses could not be paired for individual comparison.

Baseline questionnaire responses demonstrated very limited previous exposure to bedside echocardiography. Most participants reported either no prior experience or having observed bedside echocardiography only once or twice in clinical practice. Students commonly identified a desire to gain a practical understanding of ultrasound principles, image acquisition, and interpretation, with several expressing uncertainty regarding the clinical applications of POCUS Echo.

Quantitative outcomes

Confidence

Self-reported confidence improved following participation in the teaching programme. Mean total confidence scores increased from 3.67/15 before teaching (median, 4) to 9.86/15 after teaching (median, 10), representing an increase of over six points on the 15-point confidence scale (Figure 2).

**Figure 2:**
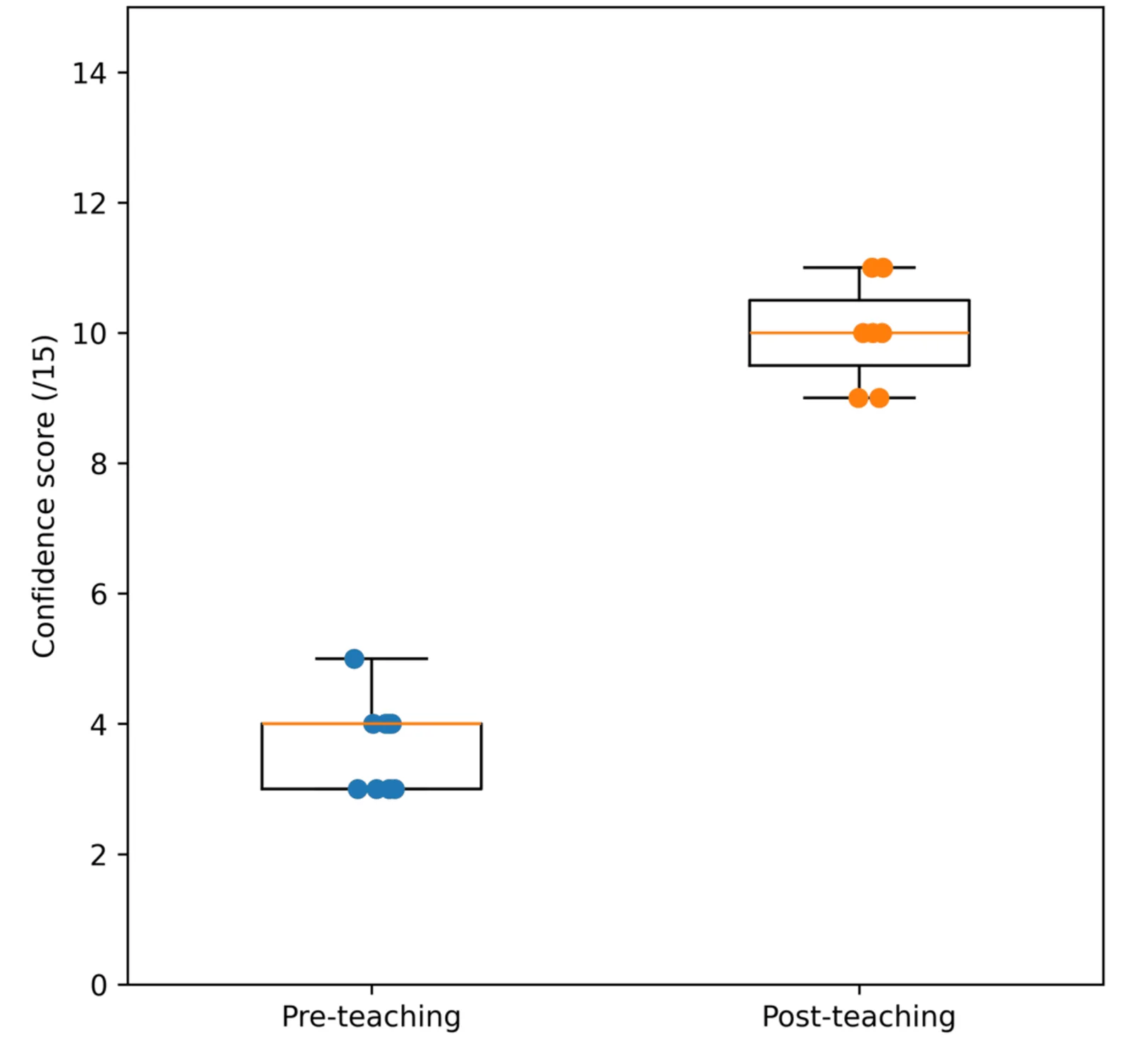
Self-reported confidence scores before and after the simulation-based bedside echocardiography teaching programme. Differences between pre-teaching (*n* = 9) and post-teaching (*n* = 7) scores were assessed using the Mann-Whitney U test (*U* = 0.0, *P* < 0.001).

A Mann-Whitney U test demonstrated a statistically significant difference in confidence scores between the pre-teaching and post-teaching groups (*U* = 0.0, *P* < 0.001).

Improvements were observed across all three assessed domains of confidence, including recognition of life-threatening echocardiographic pathology, probe positioning for standard cardiac views, and understanding of basic cardiac ultrasound views.

Knowledge

Knowledge assessment scores also improved following the intervention. Mean quiz scores increased from 4.22/5 before teaching to 4.71/5 after teaching. Most students correctly identified the indications for bedside echocardiography, recognised the role of the subcostal view in detecting pericardial effusion, and understood the use of focused echocardiography in identifying reversible causes of cardiac arrest. Descriptive knowledge assessment scores before and after the intervention are presented in Table 1.

**Table 1:** Knowledge assessment scores before and after the simulation-based bedside echocardiography teaching session.

Outcome	Pre-teaching (*n* = 9)	Post-teaching (*n* = 7)
Mean score (/5)	4.22	4.71
Median score (/5)	4	5
Minimum	3	4
Maximum	5	5

As baseline knowledge scores were already high, only modest improvements were observed in objective knowledge assessment compared with the larger improvements seen in self-reported confidence.

Qualitative feedback

All participants provided positive feedback regarding the educational intervention. Three principal themes emerged from the free-text responses.

Clinical Relevance

Students consistently described the session as directly applicable to emergency medicine practice and early postgraduate training. Several participants commented that the teaching was particularly valuable because it focused on clinical decision-making rather than theoretical ultrasound knowledge alone. Multiple students suggested that the programme was best suited to the Preparation for Practice placement immediately before graduation.

Value of Simulation-Based Learning

Participants identified the simulation component as one of the most valuable aspects of the programme. Learners highlighted the opportunity to apply bedside echocardiography within realistic peri-arrest scenarios while undertaking a structured A-E assessment, helping to integrate ultrasound findings into clinical reasoning rather than viewing image acquisition as an isolated technical skill.

Opportunities for Further Practical Experience

Although overall feedback was highly positive, several students requested additional opportunities for hands-on scanning. Suggestions included demonstrating image acquisition on a live volunteer before the simulation and increasing the amount of supervised practical probe practice. One participant also recommended greater standardisation of team roles during the simulation scenarios.

Overall, participants rated the teaching highly for clarity of learning objectives, engagement, and pacing, with the majority awarding the maximum score for each domain. Students described the session as "really good and helpful", "engaging", and clinically relevant, with several recommending incorporation of the programme into the undergraduate Emergency Department curriculum.

## Discussion

This study evaluated the development and implementation of a simulation-based bedside echocardiography teaching programme for final-year medical students during an Emergency Department placement. The programme was feasible to deliver within the undergraduate placement and was well received by participating students. Post-session respondents reported higher confidence scores than pre-session respondents, alongside slightly higher knowledge assessment scores. Qualitative feedback suggested that students considered the teaching clinically relevant and particularly valued the opportunity to apply bedside echocardiography within realistic emergency scenarios. Given the small sample size and unpaired questionnaire design, these findings should be considered preliminary but suggest that simulation-based teaching may provide an acceptable and practical approach to introducing focused cardiac ultrasound within undergraduate medical education.

The expanding use of point-of-care ultrasound across emergency medicine, critical care and acute medical specialties has led to increasing recognition of its potential role within undergraduate medical education. However, undergraduate exposure remains inconsistent. Tarique et al. identified considerable variation in the design, delivery and assessment of undergraduate ultrasound curricula, with no widely adopted standardised approach [7]. Despite this variation, previous studies have demonstrated that medical students can acquire focused cardiac ultrasound skills following relatively short periods of structured teaching. Kobal et al. demonstrated that medical students could acquire and interpret focused cardiac ultrasound images following a course consisting of four hours of lectures and four hours of hands-on training [8]. Similarly, Elison et al. demonstrated objective improvement in image acquisition among medical students following simulator-based focused cardiac ultrasound training [9]. The findings of the present evaluation are consistent with the broader literature in demonstrating positive learner outcomes following focused ultrasound teaching, although the present study assessed confidence and knowledge rather than objective technical competence.

An important feature of the programme was the integration of echocardiography into simulated emergency scenarios rather than teaching image acquisition as an isolated procedural skill. This approach is particularly relevant when compared with the study by Russell et al., who evaluated whether medical students could integrate focused cardiac POCUS into the assessment of a simulated hypotensive patient following a short educational intervention [10]. Their findings demonstrated that students were able to perform and interpret a focused cardiac ultrasound examination and apply their findings within a clinical scenario [10]. The present programme similarly aimed to move beyond ultrasound image acquisition alone. Students were required to perform an A-E assessment, recognise deterioration and use bedside echocardiography to inform diagnosis and management within emergency presentations including cardiac arrest, pericardial tamponade and massive pulmonary embolism. Qualitative feedback suggested that students particularly valued this clinical integration, supporting the use of simulation to contextualise POCUS within the wider assessment of an acutely unwell patient.

The use of simulation is also supported by wider health professions education literature. Cook et al. demonstrated that technology-enhanced simulation is associated with improvements in knowledge, skills and behaviours when compared with no educational intervention [11]. Simulation provides an environment in which learners can practise technical skills, receive feedback and develop clinical decision-making without risk to patients. Within POCUS education, it also provides controlled exposure to clinically important pathology that students may not encounter during a short Emergency Department placement. In the present programme, simulation enabled students to integrate technical, cognitive and non-technical skills rather than practising echocardiographic views in isolation.

Knowledge assessment scores were slightly higher among post-session respondents, although the difference was less pronounced than that observed for confidence. This should be interpreted within the context of the assessment itself. Baseline knowledge scores were already high, suggesting a potential ceiling effect that limited the ability of the five-question assessment to discriminate between respondents. Previous exposure to bedside echocardiography may also have contributed to the high baseline knowledge scores, although the small sample size and limited variation in knowledge scores precluded meaningful analysis of this relationship. In contrast, baseline confidence scores were considerably lower, providing greater scope for differences to be observed between the pre- and post-session groups. Although the increase in confidence is encouraging, confidence should not be interpreted as a direct measure of competence. This distinction is important when comparing the present findings with studies such as Elison et al., which used an objective measure of image acquisition and demonstrated measurable improvement in technical performance following simulator-based training [9]. The present evaluation cannot establish whether higher confidence was accompanied by improved image acquisition or interpretation.

The relatively short duration of the programme should also be considered when interpreting these findings. Kobal et al. incorporated eight hours of focused cardiac ultrasound education [8], whereas the present intervention combined ultrasound fundamentals, practical image acquisition and emergency simulation within a two-hour session. The aim was therefore not to develop independent echocardiography competence but to introduce the clinical application of POCUS Echo before students entered postgraduate practice. The ability to incorporate the programme into an existing Emergency Department placement suggests that a shorter intervention may provide a practical approach where undergraduate curriculum time and faculty availability are limited. However, further teaching and supervised scanning would be required to develop technical competence.

This was also reflected in the qualitative feedback. Although participants responded positively to the programme, several requested additional opportunities for hands-on scanning, including demonstration on a live volunteer before simulation and increased supervised probe practice. Previous undergraduate cardiac ultrasound programmes have incorporated more extensive hands-on practice alongside theoretical teaching [8], suggesting that simulation may be most valuable as one component of a broader ultrasound curriculum rather than a substitute for supervised image acquisition. A future iteration of the programme could therefore retain the emergency simulation component while increasing opportunities for practical scanning.

A strength of this evaluation was that the teaching programme was developed following a pilot needs assessment, allowing learner feedback to inform curriculum design before implementation. The programme combined theoretical teaching, practical image acquisition and scenario-based simulation within a single two-hour session. The use of both quantitative and qualitative evaluation also provided information on respondent confidence and knowledge alongside students' experiences of the programme. Furthermore, the focus on final-year students immediately before postgraduate practice may provide an opportunity to contextualise POCUS within clinical decision-making at a stage when learners are preparing to assume greater responsibility for acutely unwell patients.

Several limitations should be acknowledged. The study was conducted at a single institution with a small sample size, limiting the generalisability of the findings and the conclusions that can be drawn regarding educational effectiveness. Engagement was affected by pre-arranged teaching commitments within students' timetables and the proximity of final examinations. Although the programme was feasible to deliver, simulation-based ultrasound teaching required greater logistical preparation than conventional didactic teaching, including room bookings, faculty availability, access to ultrasound equipment and recruitment of a model. These resource requirements should be considered when assessing the feasibility of implementing similar programmes on a larger scale. Participation was also voluntary and therefore susceptible to selection bias, as students with an existing interest in emergency medicine or ultrasound may have been more likely to attend.

Importantly, pre- and post-session questionnaires were completed anonymously and could not be paired. The observed differences therefore represent differences between the pre-session and post-session respondent groups and cannot establish individual learner improvement or be attributed directly to the teaching intervention. Outcomes were also measured immediately following the session, preventing assessment of retention over time or translation into clinical performance. Furthermore, self-reported confidence should not be considered a surrogate for competence. This represents an important distinction from studies that have incorporated objective measures of image acquisition and interpretation [8,9]. These limitations mean that the findings should primarily be interpreted as evidence of the feasibility and acceptability of the programme, alongside preliminary differences in learner-reported outcomes, rather than evidence of intervention efficacy.

Future evaluation should include larger cohorts and, where possible, matched pre- and post-session responses to allow assessment of individual learner progression. Objective assessments of image acquisition and interpretation would provide a more robust measure of educational outcomes and allow comparison with previous studies demonstrating measurable acquisition of focused cardiac ultrasound skills [8,9]. Longitudinal follow-up could establish whether learning is retained during Foundation training, while evaluation across multiple sites would help determine whether the programme is feasible within different undergraduate and Emergency Medicine teaching environments.

Overall, this preliminary evaluation suggests that a simulation-based POCUS Echo teaching programme can be incorporated into an undergraduate Emergency Department placement and is acceptable to participating final-year medical students. The higher confidence scores observed among post-session respondents and positive qualitative feedback are consistent with previous undergraduate ultrasound literature [7-10]. However, unlike studies incorporating objective assessment of focused cardiac ultrasound skills [8,9], the present evaluation did not assess procedural competence. The small sample size and unpaired design also preclude conclusions regarding educational efficacy or individual learner improvement. Further evaluation in larger cohorts using paired responses, objective measures of ultrasound competence and longitudinal follow-up is required before conclusions can be drawn regarding the effectiveness of this approach.

## Conclusions

This preliminary evaluation suggests that a simulation-based bedside echocardiography teaching programme is feasible to deliver within an undergraduate Emergency Department placement and acceptable to participating final-year medical students. Students particularly valued the opportunity to apply focused echocardiography within realistic emergency scenarios, while post-session respondents reported higher confidence scores than pre-session respondents. However, the small sample size and unpaired questionnaire design mean that these findings cannot demonstrate individual learner improvement or establish the efficacy of the intervention.

The programme will be repeated with the next academic cohort to enable evaluation in a larger group. Future studies should incorporate paired pre- and post-session responses, objective measures of ultrasound competence and longitudinal follow-up to assess knowledge and skill retention. These findings provide a preliminary basis for further evaluation of simulation-based POCUS Echo teaching and its potential role within undergraduate medical education.

## Figures and Tables

**Figure 3:**
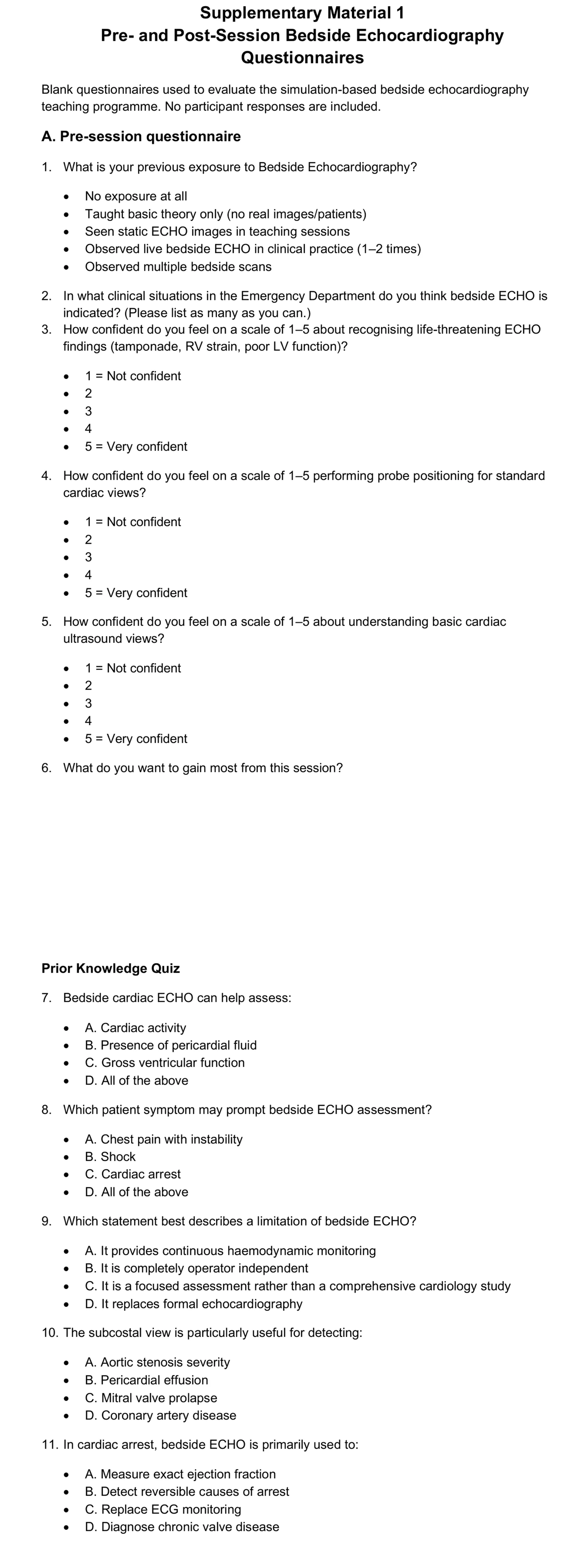
Pre-session questionnaire.

**Figure 4:**
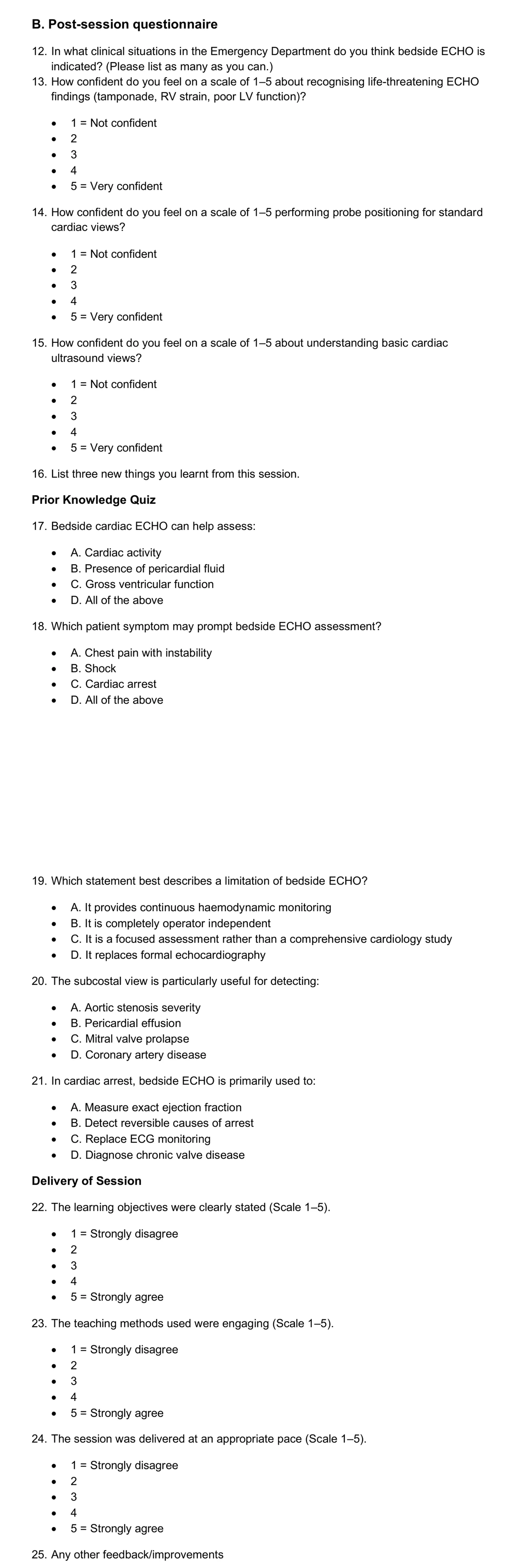
Post-session questionnaire.

## Appendices

Appendix A

Appendix B
